# INTRODUCTION **Disrupting the Status Quo:** Building Equitable Access to HIV PrEP in the US through Innovative Financing

**DOI:** 10.1017/jme.2022.29

**Published:** 2022

**Authors:** Jeremiah Johnson, Amy Killelea, Derek T. Dangerfield, Chris Beyrer, Joshua M. Sharfstein

**Keywords:** HIV, PrEP, Equity, Drug Pricing, Federal Policy

## Abstract

This special edition of *JLME* centers on a novel proposal for a national PrEP access program with the potential to break through a failed status quo.

Over a decade ago, a new era in biomedical HIV prevention began. In 2010, the iPrEx phase three trial found that a single pill containing tenofovir disoproxil fumarate (TDF) and emtricitabine (FTC) taken daily for HIV pre-exposure prophylaxis (PrEP) could reduce the chances of contracting HIV among men who have sex with men (MSM) and transgender women.^1^ Further data ultimately showed that with consistent adherence PrEP provided up to a 99% reduction in HIV acquisition through sex.^2^ Soon after, other federally sponsored studies showed that PrEP was 74% effective in reducing risk among people who inject drugs.

Along with the hopeful findings of HPTN 052 in 2011 and the eventual determination that among people living with HIV an undetectable viral load made HIV sexually “untransmittable” (a concept dubbed U=U by community advocates),^3^ it appeared that a pandemic that had raged visibly since 1981 might finally be conquered through scientific innovation. Along with dramatically expanded healthcare access through the passage of the Affordable Care Act in 2010, it seemed that healthcare coverage barriers to biomedical HIV prevention were simultaneously being addressed.

By 2012, PrEP was FDA-approved and community activists in New York, San Francisco, and Washington State began to call for an end to the epidemic.^4^ In New York, this culminated in then Governor Andrew Cuomo’s 2014 support of a plan for the state that would seek to bring new infections below epidemic levels.^5^ Soon similar calls from advocates for widespread access to the innovations that could dramatically reduce transmission were coming from Fulton County, GA, Houston, Washington, D.C., and spreading quickly through other heavily impacted jurisdictions. Eventually, those same calls would influence the *Ending the HIV Epidemic: A Plan for America* (EHE) initiative announced by President Trump’s administration in 2019.^6^

However, as we head toward the 12th anniversary of the iPrEx findings and the 10th anniversary of the FDA approval of PrEP, the feasibility of ending the epidemic is questionable, and PrEP remains out of reach for most people who could best benefit. There are massive disparities in PrEP initiation and adherence by income, race, ethnicity, age, gender, and region. The status quo for PrEP provision—hindered by ongoing gaps in healthcare coverage and centered around high-cost medications and fractured delivery systems — is not on track to meet EHE targets, particularly at a time when prevention and testing services have been substantially disrupted during the COVID-19 pandemic.^7^

It’s time for a different approach. This special edition of the *Journal of Law, Medicine & Ethics (JLME)* centers on a novel proposal for a national PrEP access program.^8^ The proposed program would streamline access to PrEP medications for people covered by Medicaid or without insurance coverage, enhance clinical care for PrEP, and create a new network of PrEP access points in communities.

It’s time for a different approach. This special edition of *JLME* centers on a novel proposal for a national PrEP access program. The proposed program would streamline access to PrEP medications for people covered by Medicaid or without insurance coverage, enhance clinical care for PrEP, and create a new network of PrEP access points in communities.

Since finalizing the content for this supplement, President Biden’s FY 2023 budget proposal included funding for a national PrEP program. The administration has proposed a 10-year $9.8 billion mandatory spending program that, according to the Department of Health and Human Services’ Operating Plan would “create a financing and delivery system for PrEP” and “guarantee access to PrEP at no cost; eliminate costs for essential associate services; and establish a network of providers in underserved communities that provide culturally and linguistically appropriate services.”^9^ It will be up to Congress to invest in this visionary program, and advocates are presently exploring pathways to turn the proposal into a reality.

As outlined in the proposal published here and alluded to in the Biden proposal, to maximize the benefits of such a program, the federal government would purchase and distribute PrEP medications both on site in clinical settings and through a large pharmacy network, leveraging newly cheap generic TDF/FTC availability. A similar approach to national negotiation and contracts with major labs would allow the Centers for Disease Control and Prevention (CDC) to cover required lab work through a widely available network. Finally, a federal grant program would empower and require health departments to develop an expansive network of traditional and nontraditional PrEP providers. In the plan, existing CDC-sponsored HIV prevention grantees would receive additional funding to improve capacity for engaging in the national program. Among other elements, the initiative would expand provider training, increase community awareness of both the national program and PrEP more generally through campaigns, and specifically leverage telehealth to ensure that even non-clinical sites, such as homeless shelters or domestic violence programs, can participate and serve their key populations.

The papers in this special *JLME* supplement provide key context and address important considerations for such a strategy. Beyrer, McCormack, and Grulich argue that PrEP is a high-impact and necessary tool to prevent HIV incidence for individual- and public health.^10^ The authors review successful examples that demonstrate how coordinated, comprehensive PrEP access initiatives can very successfully and rapidly increase uptake in global contexts, including Australia, England, and the U.S. Additionally, Sharfstein, Conti, and Gee emphasize the benefits of national coordination of a PrEP access strategy, citing the benefits of governmental leadership in efforts to rapidly implement COVID vaccine access.^11^ Their piece also highlights other effective examples of governmental price negotiation and centralized distribution, drawing lessons from the Vaccines for Children program and Louisiana’s “subscription model” approach to procuring expensive hepatitis C medications at significantly reduced cost.

Two articles help frame current challenges and trends in national PrEP coverage and access. Ballreich, Levengood, and Conti, drawing from available data on private insurance and Medicaid-covered PrEP prescriptions from the first quarter of 2021, show that generic forms of PrEP have had limited uptake and have yet to contribute to overall expansions in use of PrEP.^12^ Siegler and Sullivan estimate that 48.9% of Americans are not covered by the recent U.S. Preventive Services Task Force “A” rating for PrEP and the requirement that payers cover all related services without cost sharing.^13^ Using an implementation science framework, they also outline several key programmatic considerations for any proposal seeking to increase coverage and access for vulnerable communities.

Bridging this macro-level context with a discussion on equitable access, Farrow explains how high PrEP costs lead to downstream pressures that restrict use for vulnerable communities, even in cases where a patchwork of coverage programs is supposedly available for uninsured and underinsured individuals.^14^ Despite arguments that costs do not matter so long as there is coverage, Farrow elaborates on ways to withhold and discourage uptake of a pricey preventive health intervention.

Two commentaries center around patients and vulnerable communities who are most in need of PrEP. Malebranche and colleagues emphasize that program flexibility must be the hallmark of any national approach to coverage and access for uninsured and Medicaid-covered individuals since “sexual risk is contextual and fluid.”^15^ The authors call specific attention to end user interfaces that are easy and intuitive, significantly broader prescribing networks, and prescribing flexibility for mail order and 90-day scripts. Johnson, Radix, Copeland, and Chacon walk through specific considerations for transgender and gender diverse, Black, and Latinx communities who stand to disproportionately benefit from a national PrEP plan.^16^

A national PrEP plan in the U.S. can only work if there is effective coordination with existing programs and resources. Seiler, Heyison, Dwyer, Karacuschanksy, Organick-Lee, and Horton provide specific guidance on how to best ensure coordination between a national PrEP plan and Medicaid, the backbone of access to care for millions of Americans.^17^ One of their key points is that state Medicaid programs should avoid seeing such a new program as ending their own responsibility to appropriately cover and promote PrEP through the establishment of an adequate prescriber network. Comer and Fernández discuss the key role of state and local health departments in a national PrEP plan.^18^ They make the case for strong partnerships and resource sharing with key populations and community-led organizations to promote true equitable access.

Since the announcement of the federal initiative to end the HIV epidemic, the CDC has called repeatedly for “disruptive innovation.” For PrEP access, the moment for disruptive innovation is now. President Biden’s proposal has raised the bar on what is possible for PrEP, and advocates and members of Congress are now charged with establishing a program that opens the flood gates on PrEP access. This issue of the *Journal of Law, Medicine & Ethics* is an important contribution to this effort.

## References

[1] R.M. Grant , et al., “Preexposure Chemoprophylaxis for HIV Prevention in Men Who Have Sex with Men,” New England Journal of Medicine 36 (2010): 2587–2599. 10.1056/NEJMoa1011205 PMC3079639 21091279

[2] Centers for Disease Control and Prevention (CDC), “Oral Daily Pre-Exposure Prophylaxis (PrEP) for HIV-Negative Persons,” *available at* <https://www.cdc.gov/hiv/risk/estimates/preventionstrategies.html#anchor_1562942347 (last March 8, 2022).

[3] Prevention Access Campaign, “Our History,” Prevention Access Campaign, *available at* <https://preventionaccess.org/about/> (last visited March 8, 2022).

[4] ACT NOW:END AIDS, “About Us,” ANEA, *available at* <https://anea.org/about-us> (last visited March 8, 2022).

[5] A. Hartocollis , “Cuomo Plan Seeks to End New York’s AIDS Epidemic,” New York Times, June 28, 2014, *available at* <https://www.nytimes.com/2014/06/29/nyregion/cuomo-plan-seeks-to-end-new-yorks-aids-epidemic.html> (last visited March 8, 2022).

[6] L. Dawson and J. Kates , “The U.S. Ending the HIV Epidemic (EHE) Initiative: What You Need to Know,” Kaiser Family Foundation, February 9, 2021, *available at* <https://www.kff.org/hivaids/issue-brief/the-u-s-ending-the-hiv-epidemic-ehe-initiative-what-you-need-to-know/> (last March 8, 2022).

[7] S. Varney , “Strides Against HIV/AIDS in the US Falter as Resources Diverted to Fight COVID-19,” NPR, April 21, 2021, *available at* <https://www.npr.org/sections/health-shots/2021/04/21/988813979/strides-against-hiv-aids-in-the-u-s-falter-as-resources-diverted-to-fight-covid> (last visited March 8, 2022).

[8] A. Killelea et al., “Financing and Delivering Pre-Exposure Prophylaxis (PrEP) to End the HIV Epidemic,” Journal of Law, Medicine & Ethics 50, no. 2, Suppl. (2022): 8–23. 10.1017/jme.2022.30 PMC9341207 35902089

[9] U.S. Dept. of Health and Human Services, “Fiscal Year 2023 Justification of Estimates for Appropriations Committees,” HHS, *available at* <https://www.hhs.gov/sites/default/files/fy2023-gdm-operating-plan.pdf> (last visited May 17, 2022).

[10] C. Beyrer , “Pre-Exposure Prophylaxis for HIV Infection as a Public Health Tool,” Journal of Law, Medicine & Ethics 50, no. 2, Suppl. (2022): 24–28. 10.1017/jme.2022.31 PMC9341191 35902085

[11] J. Sharfstein et al., “From COVID Vaccines to HIV Prevention: Pharmaceutical Financing and Distribution for the Public’s Health,” Journal of Law, Medicine & Ethics 50, no. 2, Suppl. (2022): 29–31. 10.1017/jme.2022.32 PMC9341206 35902083

[12] J. Ballreich , “Opportunities and Challenges of Generic Pre-Exposure Prophylaxis Drugs for HIV,” Journal of Law, Medicine & Ethics 50, no. 2, Suppl. (2022): 32–39. 10.1017/jme.2022.33 PMC9341204 35902088

[13] A. Siegler and P. Sullivan , “The PrEP Laboratory Service Gap: Applying Implementation Science Strategies to Bring PrEP Coverage to Scale in the United States,” Journal of Law, Medicine & Ethics 50, no. 2, Suppl. (2022): 40–46. 10.1017/jme.2022.34 PMC9341189 35902081

[14] K. Farrow , “The Downstream Impacts of High Drug Costs for PrEP Have Hindered the Promise of HIV Prevention,” Journal of Law, Medicine & Ethics 50, no. 2, Suppl. (2022): 47–50. 10.1017/jme.2022.35 PMC9341201 35902087

[15] D. Malebranch , “Implementing a National PrEP Program: How Can We Make It Happen?” Journal of Law, Medicine & Ethics 50, no. 2, Suppl. (2022): 51–54. 10.1017/jme.2022.36 PMC9341202 35902086

[16] J. Johnson , “Building Racial and Gender Equity into a National PrEP Access Program,” Journal of Law, Medicine & Ethics 50, no. 2, Suppl. (2022): 55–59. 10.1017/jme.2022.37 PMC9341199 35902091

[17] N. Seiler et al., “Navigating the Intersection of PrEP and Medicaid,” Journal of Law, Medicine & Ethics 50, no. 2, Suppl. (2022): 60–63. 10.1017/jme.2022.38 PMC9341193 35902082

[18] C. Comer and R. Fernández , “Health Department and PrEP: A Missed Opportunity for Public Health,” Journal of Law, Medicine & Ethics 50, no. 2, Suppl. (2022): 64–68. 10.1017/jme.2022.39 PMC9341197 35902080

